# Does GPER Really Function as a G Protein-Coupled Estrogen Receptor *in vivo*?

**DOI:** 10.3389/fendo.2020.00148

**Published:** 2020-03-31

**Authors:** Jing Luo, Dongmin Liu

**Affiliations:** ^1^Department of Nutrition, School of Public Health, Sun Yat-sen University, Guangzhou, China; ^2^Department of Human Nutrition, Foods and Exercise, College of Agricultural and Life Sciences, Virginia Tech, Blacksburg, VA, United States

**Keywords:** GPR30, GPER, estrogen receptor, estrogen, physiological role

## Abstract

Estrogen can elicit pleiotropic cellular responses via a diversity of estrogen receptors (ERs)—mediated genomic and rapid non-genomic mechanisms. Unlike the genomic responses, where the classical nuclear ERα and ERβ act as transcriptional factors following estrogen binding to regulate gene transcription in estrogen target tissues, the non-genomic cellular responses to estrogen are believed to start at the plasma membrane, leading to rapid activation of second messengers-triggered cytoplasmic signal transduction cascades. The recently acknowledged ER, GPR30 or GPER, was discovered in human breast cancer cells two decades ago and subsequently in many other cells. Since its discovery, it has been claimed that estrogen, ER antagonist fulvestrant, as well as some estrogenic compounds can directly bind to GPER, and therefore initiate the non-genomic cellular responses. Various recently developed genetic tools as well as chemical ligands greatly facilitated research aimed at determining the physiological roles of GPER in different tissues. However, there is still lack of evidence that GPER plays a significant role in mediating endogenous estrogen action *in vivo*. This review summarizes current knowledge about GPER, including its tissue expression and cellular localization, with emphasis on the research findings elucidating its role in health and disease. Understanding the role of GPER in estrogen signaling will provide opportunities for the development of new therapeutic strategies to strengthen the benefits of estrogen while limiting the potential side effects.

## Introduction

Estrogen, more specifically, 17β-estradiol (E2), is a female sex hormone, which is essential for not only the development of the female reproductive organs but also the secondary sex characteristics (1). In addition, this hormone plays a critical role in the development and function of the male reproductive tract (2). Moreover, E2 plays important physiological roles in almost every part of the body, including the nervous system (3), immune system (4, 5), skeletal tissue (6, 7), musculature (8–11), as well as the endocrine system (12, 13). E2 exerts the comprehensive physiological effects by interacting with estrogen receptors (ERs) and subsequently, activating various signaling cascades that extend from seconds to hours (14, 15). In this review, we provide a brief overview of estrogen signaling and describe the characteristics of its receptors, emphasizing on GPR30, presumably a G protein-coupled ER (GPER). We focus on discussing studies aimed at elucidating the potential physiological and pathological roles of GPER in regards to its estrogen binding properties and in mediating the actions of E2 *in vivo*. In addition, this review also summarizes recent research that supports E2-independent effects of GPER in various tissues.

## Estrogen Receptors

Steroid hormones are synthesized in the ovaries (E2, progesterone), testes (androgens, testosterone), and adrenal glands (cortisol, androgens). E2 is a critical steroid hormone that was originally believed (in the 1960's) to exert its physiological effects through a nuclear ER, later termed as ERα, which was identified in the rat uterus (16–18). About three decades later, the first ERα knockout mouse model was generated (19). The second ER, ERβ, was identified in the rat prostate in 1996 (20). ERs are ligand-regulated nuclear transcriptional factors that are believed to mediate a wide array of biological actions of E2.

Besides these classical nuclear ERs, which can initiate transcriptional events in the promoter regions of target genes, E2 is also reported to engage in rapid non-genomic signaling events (21, 22). Several studies have shown that E2 triggers a variety of intracellular signaling events, including mobilization of intracellular calcium in MCF-7 breast cancer cells (23), production of cyclic adenosine monophosphate (cAMP) in primary rat uterine cells (24), activation of mitogen-activated protein kinases p38 in MCF-7 cells and ROS17/2.8 rat bone cell line (25, 26), and activation of extracellular signal-regulated kinase 1/2 (ERK 1/2) in human neuroblastoma cells (27). The underlying mechanisms for E2 exerting these rapid cellular actions appear to be complex that may involve ERs, the variants of ERα, and unknown E2 receptors (22, 28). Cellular signal transduction can occur as a result of E2 activating G proteins, which then lead to the modulation of downstream cellular pathways (29–31). Thus, a potential role for G protein-coupled receptors (GPCRs), which utilize E2 as ligand, has been proposed as an important route through which E2 exerts cellular functions.

## GPER, an Atypical G Protein-Coupled Receptor

### Discovery of GPER

As early as the 1960–1970s, two independent studies reported the rapid cellular effects of E2 on cAMP synthesis (32) and calcium mobilization (33). These acute effects evoked by E2 are transmitted through enzymes and ion channels via the activation of membrane-associated ERs that may not involve transcription, which are thereby referred as non-genomic or extra-nuclear signaling pathways (34, 35). In 1997, a novel seven transmembrane-domain GPCR, named GPR30, was first identified and cloned (36), which showed high sequence homology to the interleukin 8 receptor and the angiotensin II receptor type 1 (37, 38). Therefore, it was initially speculated that the endogenous ligand activating GPR30 is a chemokine or peptide (37, 39). However, chemokines and/or peptides failed to evoke responses in GPR30 transfected cells (37, 39), suggesting that GPR30 might be an orphan GPCR without cognate endogenous ligands. In 2004, Maggiolini et al. performed gene expression analysis of SKBr3 cells lacking ERs. The results indicated that the proto-oncogene c-fos was upregulated in response to E2. Interestingly, the upregulation of c-fos by E2 was blocked when the endogenous GPR30 expression was silenced (40). In another study that used breast cancer cell lines, GPR30 expression was positively correlated with ERα expression, suggesting these two receptors might be regulated by the same regulatory mechanism or transcription factors (36). The orphan fate of GPR30 reached a turning point in 2005 (41). Two independent research groups provided data demonstrating that E2 directly binds to GPR30, which thus acts as a membrane-bound ER (30, 31). In 2007, the physiological role of GPR30 *in vivo* was first examined in rats (42). The results showed that administration of E2 induced GPR30 expression and attenuated hepatic injury via protein kinase A (PKA)-mediated mechanism in rats. Consistently, knockdown of GPR30 but not ERα attenuated the E2-dependent activation of PKA in hepatocytes isolated from rats. Therefore, GPR30 was officially named as GPER by the International Union of Basic and Clinical Pharmacology in 2007 (43). The characteristics of all three known ERs are summarized in Table 1.

**Table 1:** Characteristics of ERs (44–52).

**ER characteristics**	**ERα**	**ERβ**	**GPER**
Category	Nuclear steroid hormone receptor superfamily		G protein-coupled receptor superfamily
Location	Nucleus	Nucleus	Membrane-associated
Size	595 aa	530 aa	375 aa
Numbers of isoforms	3	5	1
Chromosome region	6q25.1	14q23.2	7p22.3
Structure	DNA-binding domain, ligand-binding domain, N-terminal domain		7 transmembrane α-helical regions, 4 extracellular and 4 cytosolic segments
Distribution in tissues	Hypothalamus, hippocampus, testes, ovary, endometrium, uterus, prostate, kidney, liver, breast, epididymis, muscle, adipose tissue	Testes, ovary, prostate, vascular endothelium, bladder, colon, adrenal gland, pancreas, muscle, adipose tissue	Central and peripheral nervous system, uterus, ovary, mammary glands, testes, pancreas, kidney, liver, adrenal and pituitary glands, cardiovascular system, adipose tissue

With the discovery of GPR30 as a novel ER (GPER), growing evidence has emerged to describe the rapid action of E2 via GPER (15, 30, 36, 53). A search in PubMed in January 2020 with the keywords “GPR30 or GPER and estrogen” yielded 1,280 publications since 1997, with 88.6% (1, 54) published during the past decade. This area has attracted a surge of interest recently and represents one of the most active area in the field of E2 research.

### GPER Expression in Tissue

The expression of GPER protein is not only restricted to E2-responsive tissues, as originally speculated. It is also present in many other tissues in humans (36–39, 55, 56) and rodents (57–62), such as brain, placenta, lung, liver, prostate, ovary, pancreatic islets, adipose tissue, vasculature, muscle, skeleton, as well as immune cells (63, 64). Interestingly, it appears that the expression pattern of GPER is age-, species-, gender-, or tissue-dependent. For example, the mRNA expression of GPER in skeletal muscle tends to be higher in premenopausal women compared to post-menopausal women (65). In mouse skeletal muscle, GPER mRNA abundance is almost 4-fold greater in females than that in males (57), with greater expression of GPER mRNA in female soleus than in extensor digitorum longus muscle (EDL) (66). GPER is also highly expressed in human bone tissues, and thus it may mediate the action of E2 on preserving bone density (67), suggesting a potential therapeutic strategy to prevent or alleviate menopausal osteoporosis by targeting GPER. Moreover, a high density of GPER was detected in the brain of hamster, including hypothalamus, thalamus, cerebellum, and amygdala, and the expression pattern of GPER behaved in a sexually dimorphic fashion in both young (post-natal 7 days) and adult (post-natal 60 days) animals (68). The gene expression of GPER was significantly higher in adult female hypothalamus than that of adult male, whereas the opposite expression pattern was observed in thalamus in young hamster. Similarly, the expression pattern of GPER mRNA displayed contrary trend in cerebellum and amygdala areas in young hamster between male and female (68). However, it is presently unclear whether GPER shows a similar expression pattern in humans. Interestingly, GPER expression is developmentally regulated. In the mammary gland, GPER abundance is lower in the elongating ducts during puberty and then increases through periods of sexual maturity (15). In the cartilage of the human growth plate, GPER expression decreases as puberty progresses in both genders (69). Studies have shown that GPER expression level in mammary ductal epithelia is dependent on estrous cycle (15), and consistently, the highest GPER mRNA expression level was found on day 3 of estrous cycle and then declined to the lowest level on day 12 in equine endometrium (70). Results from another study examining GPER expression in hamster ovarian cells during estrous cycle exhibited similar pattern that GPER mRNA and protein abundance reached the peak levels on day 3 of estrous cycle and decreased on day 4. These findings are very important, as they provide a basis for investigating the physiological or pathological roles of GPER including cancer development, immune regulation, and reproductive, cardiovascular, as well as metabolic functions (64, 71).

### GPER Localization in Cells

GPER is a seven transmembrane GPCR and therefore it is presumed to be located on the plasma membrane (72) as are most GPCRs (30, 73). Indeed, it has been shown that GPER induces signaling via activation of Gαs or Gαi (15, 30), strongly suggesting that this receptor is associated with the plasma membrane. Interestingly however, several studies provide evidence showing that a larger fraction of total cellular GPER is localized in intracellular compartments. Revankar et al. used fluorescent E2 derivatives (E2-Alexas) to visualize the extra- and intracellular binding properties of GPER in COS-7 (monkey kidney fibroblast) cells. Surprisingly, the confocal images revealed that E2-Alexas failed to label the plasma membrane but predominantly bound to endoplasmic reticulum (31). In addition, E2-Alexas- or antibody-stained GPER is also co-localized in the Golgi apparatus and nuclear membrane in GPER expressing cancer cell lines (31). Similarly, the predominant intracellular staining pattern of GPER was also observed in human umbilical vein endothelial cells (74), vascular smooth muscle cells (74), and pancreatic islet cells (75, 76). Intriguingly, fluorescent microscopy and western blotting evidenced that GPER was present in mitochondria in undifferentiated C2C12 myoblasts, but was found in cytoplasm in differentiated C2C12 myotubes that modulates E2 actions (77). However, some other studies reported that GPER is mainly localized to the plasma membrane of uterine epithelia (78), myometrium (79), renal epithelia (80–82), and hippocampal neurons (73, 83), though an intracellular expression of GPER has also been reported in neurons (60). Therefore, the cellular distribution of GPER apparently varies depending on species, tissue, and cell types. Interestingly, several studies indicated that GPER is activated intracellularly, which then diffuses across cell membranes and initiates cellular signaling (31, 84, 85). These results indicate that GPER is an atypical GPCR, and its intracellular location may dynamically change in response to specific environmental cues and also could be tissue-dependent. Thus, a role for GPER as a plasma membrane-based ER is still controversial, and the exact mechanism by which GPER acts in response to E2 remains elusive.

### GPER Ligands

As discussed above, studies utilizing E2-Alexa or a fluorescent derivative of E2 demonstrated intracellular localizations of GPER (31, 86). Measurement of steroid binding to membrane-associated receptors is challenging because of the lipophilic nature of steroids and relatively low levels of membrane proteins that cause high background binding. Nevertheless, results from ligand binding assays demonstrated that GPER is a specific receptor for E2 with estimated binding affinities of 3–6 nM (30, 31), which is however much lower as compared with its binding affinities for classical ERs that are in the range of 0.1–1.0 nM (87).

In addition to E2, compounds with estrogenic activity can be found in a large variety of natural sources such as plants (e.g., soy) and fungi (88). With the rapid development of synthetic estrogenic substances, it is not surprising that a large number of estrogenic compounds have been shown to interact with GPER. Tamoxifen, for instance, is a well-known selective ER modulator and found to act as a GPER agonist (31, 89). Interestingly, stimulation with 4-hydroxytamoxifen, the active metabolite of tamoxifen, failed to activate PI3K in ERα positive cells but did activate PI3K in GPER expressing cells (86). Another widely used selective ERα/β antagonist, ICI182,780 (ICI), was also shown to bind to GPER (30) and activate this receptor (90). In GPER-transfected MDA-MB-231 breast cancer cells (ERα-deficient), ICI can activate ERK1/2 (90), confirming its effect as a GPER agonist. Consistently, another recent study demonstrated that raloxifene, a selective ER modulator, also elicited cellular response via GPER in ERα-deficient endometrial carcinoma Hec50 cells (91). In addition, numerous synthetic estrogenic compounds have been shown to bind and/or activate GPER, including zearalonone, non-phenol, kepone, p, p′-DDT, o, p′-DDE, 2, 2′, 5′, -PCB-4-OH (92), and bisphenol A (93, 94). Finally, several lines of research have demonstrated the agonistic actions of some plant-derived polyphenolic compounds toward GPER, including genistein (40, 92, 95, 96), quercetin (40), equol (97), resveratrol (98), oleuropein, hydroxytyrosol (99), and daidzein (100). However, it should be noted that the results from these studies were obtained exclusively from *in-vitro*-based assays using cancer cells or clonal cells with artificially over-expressed GPER, and whether and how they exert estrogenic effects as well as the target tissue *in vivo* are still unknown. Hence advancing the field of GPER research using these estrogenic compounds is fraught with complications. Fortunately, a highly selective GPER agonist, G-1, was synthesized in 2006 (101) and further studies of GPER action are greatly facilitated by this compound.

G-1 showed high binding affinity for GPER (Kd = 10 nM) without binding to ERα/β at concentrations as high as 10 μM (102). Three years later, a subsequent study identified a highly selective GPER antagonist, G15, with a similar structure as G-1 but lacking the ethanone moiety (103), which displayed a minimal binding to ERα/β (Kd >10 μM) (104). Another GPER specific antagonist G36 was generated to restore the steric bulk of G-1 and the ER counter selectivity (102). These selective modulators of GPER have been used in over 200 studies to evaluate GPER actions in a variety of cellular and animal models. More recently, a first peptide GPER ligand corresponding to part of the hinge region/AF2 domain of the human ERα was identified, which acts as an inverse agonist of GPER to suppress mitogenic signaling and inhibit breast cancer cell growth (105, 106). In addition, two novel GPER specific agonists, GPER-L1 and GPER-L2, were synthesized in 2012 with binding affinities of ~100 nM (107). The same year, a synthetic molecule, named as MIBE, was reported to bind and block both ERα and GPER activity in breast cancer cells (108). Recently, a small molecule with high binding selectivity to ERα/β over GPER, termed AB-1, was generated, which may further aid distinguishing the roles of ERα/β and GPER in E2 signaling (109). Intriguingly, the widely used ERα specific agonist, propyl pyrazole triol (PPT), has been reported to act as GPER agonist at concentrations as low as 10–100 nM. On the contrary, the ERβ specific agonist, diarylpropionitrile (DPN), had no effect on GPER at concentrations up to 10 μM (91). Therefore, the results from studies regarding the use of these compounds aimed at modulating ER actions should be interpreted carefully with respect to the concentrations of these compounds.

## GPER in Health and Disease

**Figure 1:**
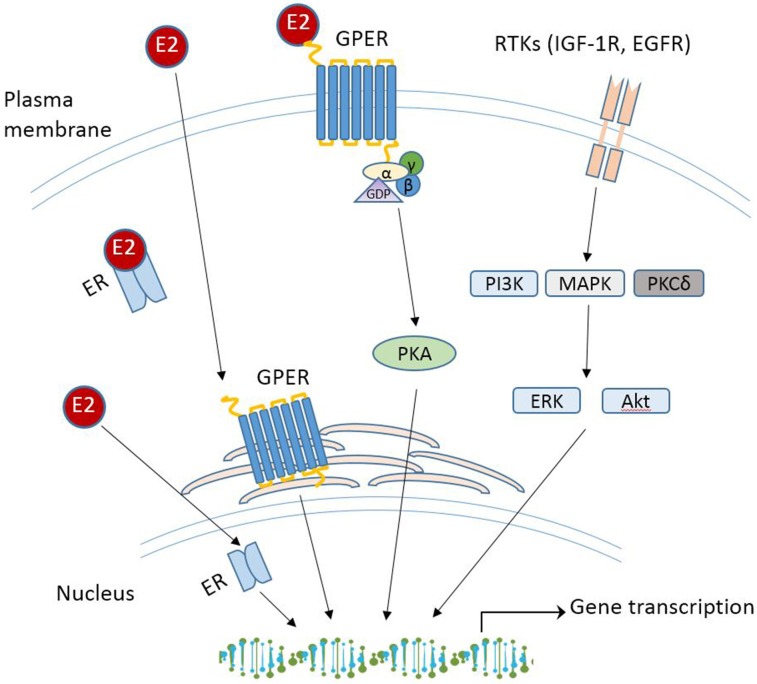
Schematic overview of E2 signaling pathways. RTK, receptor tyrosine kinases; IGF-1R, insulin-like growth factor 1 receptor; EGFR, epidermal growth factor receptor; PI3K, phosphoinositide 3-kinase; MAPK, mitogen-activated protein kinases; PKCδ, protein kinase C-delta; Akt, protein kinase B.

With the increasing spectrum of research on GPER *in vitro*, many critical questions remain: what is the physiological role of GPER? Does GPER really serve as a GPER and act independently or collaborate with the classical ERs? Will drugs targeting GPER be more effective than those targeting ERα/β for treatment of disease? Although GPER was officially named by the International Union of Basic and Clinical Pharmacology in 2007 (43), deciphering the physiology role(s) of GPER as a novel ER in health and disease remains challenging, which is due to the complex nature of E2-initiated cellular events that involve multiple receptors, various cellular signaling cascades, direct or indirect binding of the E2-ER complex to DNA, and regulation of gene expression. While these aspects are beyond the scope of this review, various mechanisms of E2 signaling are summarized in Figure 1. The readers can refer to other review papers on this topic [see (90, 110, 111) for more detailed information]. In the following section, recent studies regarding the physiological roles of GPER in different tissues and disease are discussed.

### GPER in Reproductive System

Since GPER is believed to be an ER, its action in the reproductive system attracted considerable attention. Early studies investigating the action of GPER were performed in various cancer cells derived primarily from reproductive tissues, including breast (30, 36, 36), ovary (112–114), endometrium (89, 115, 116), testis (117, 118), prostate (119), as well as thyroid tissues (95, 120). Since GPER was first identified and cloned in breast cancer cells, much early research has focused on exploring the role of GPER in various types of cancer, which has been reviewed thoroughly elsewhere (121–123) and will be briefly discussed in this space. For instance, Upon stimulation with E2, estrogenic compounds (e.g., genistein, hydroxytamoxifen) or selective GPER agonist G-1, GPER enhanced cancer cell proliferation in the classical-ER-negative breast cancer cells (30) and in the thyroid (95), endometrial (89), and ovarian cancer cells (113), suggesting that GPER may contribute to E2-induced cancer growth. Research by De Francesco., el al. provides novel insight into the mechanism by which activation of GPER triggers cancer cell proliferation (124). Specifically, their research demonstrated that E2 and GPER specific agonist G-1 upregulated HIF1α-dependent vascular endothelial growth factor expression in ER-negative breast cancer cells and cancer-associated fibroblasts, which led to angiogenesis and breast cancer progression as shown in a mouse xenograft model of breast cancer. Interestingly, It was shown that GPER specific agonist G-1 suppressed SKOV-3 and OVCAR-3 cell proliferation and activated caspase-dependent cell apoptosis, indicating that GPER may act as a tumor suppresser for ovarian cancer (125). In line with this finding, several reports also discovered such contrary effects of GPER in reproductive cell lines. Activation of GPER by E2 or G-1 suppressed human bladder urothelial cell proliferation via down-regulating the activation of protein-1 (AP-1) (126), which is one of the major regulators of cell proliferation (127). Similarly, in the classical ER negative human breast cancer cell lines SkBr3 and MDA-MB-231, activation of GPER by G-1 inhibited cell proliferation and induced G2 cell-cycle arrest *in vitro* and suppressed ER negative breast cancer growth *in vivo* (128). In ovarian-like granulosa tumor cells, E2 activates GPER-protein kinase C signaling, which then phosphorylates forkhead transcription factor 2 to promote cell apoptosis (129). In human, activation of GPER by G-1 enhanced contractile responses to oxytocin in the myometrium during labor (79). In addition, GPER together with ERα regulates the proliferative and/or apoptotic pathways involved in spermatogenesis via the EGFR/ERK/c-jun pathway in male rodent reproductive development (130, 131). However, the physiological relevance of these *in vitro* findings are unknown. Actually, in contrast to these *in vitro* findings, activation (101, 132) or deletion of GPER (57, 58) displayed no effect on the development of reproductive organs in mice, which is in clear contrast to the established phenotype of these animals lacking ERα or E2. This result suggests that GPER may be either not endogenously activated by E2 or not involved in mediating estrogenic actions of E2 in reproductive organs. Interestingly, studies from ovariectomized mice indicated that activation of GPER inhibited E2-induced uterine epithelial cell proliferation, which was associated with the reduced E2-stimulated ERα phosphorylation (78), Therefore, GPER inhibition of E2-stimulated cell proliferation may be mediated via suppressing the phosphorylation of ERα, which is important for various E2-stimulated transcriptional events (54), suggesting a possible “yin-yang” relationship between these two receptor. These findings demonstrate the complex roles of GPER in reproductive system and further investigation is needed.

### GPER in Cardiovascular System

Increasing evidence shows that GPER exerts cardioprotective effects. In mouse models, It was shown that global deletion of GPER increased blood pressures (57), atherosclerosis progression and systemic inflammation (74). GPER also plays a direct cardioprotective role, as mice with a cardiomyocyte-specific deletion of GPER displayed abnormal cardiac structure and impaired systolic and diastolic function (133). GPER may mediate a direct vasodilatory effect of E2 in vasculature. As a selective antagonist of the classic ERα/β, ICI exhibited agonistic action on GPER and promoted the dilation of coronary artery in porcine (134). Interestingly, acute infusion of GPER selective agonist G-1 decreased blood pressure in male rats, while long-term injection of G-1 decreased mean arterial pressure in the hypertensive ovariectomized female rats, suggesting that activation of GPER potentially protects E2-insufficient females from hypertension (135). In line with the results from using animal models, stimulation by G-1 dilated human internal mammary arteries and notably, the relaxant effects of G-1 were more potent than that of E2 in precontracted human aorta and carotid artery (136). The exact mechanism for blood pressure-lowering action of GPER is not clear. Studies from GPER knockout mice suggest that GPER can directly stimulate nitric oxide (NO) production from endothelial cells (ECs) and subsequent vessel dilatation (137, 138). Indeed, treatment with GPER selective antagonist G36 suppressed E2-induecd NO release in human ECs, whereas activation of GPER with G-1 promoted endothelial NO synthase phosphorylation (138), suggesting that GPER is at least partially responsible for NO-mediated vasodilatory action of E2. Another study demonstrated that activation of GPER inhibited endothelin-triggered vasoconstriction via reducing vascular smooth muscle cell Ca (2+) sensitivity (139). Further, GPER was shown to protect against angiotensin (Ang) II-induced hypertension through suppressing NADPH oxidase 4-dependent oxidative stress via activation of cAMP signaling pathway (140), suggesting that this receptor also exerts an antioxidant role. Therefore, GPER could potentially be a target for developing strategy to promote cardiovascular health.

### GPER in Nervous System

Estrogen has many beneficial effects in the brain, which include improving cognitive performance (141), opposing the early occurring hippocampal damage (142), increasing neuronal connectivity (143), and preventing or slowing age-related cognitive decline (144). Although these protective effects of E2 are largely attributed to the classical ERα/β, increasing evidence demonstrates that GPER also plays potential role(s) in E2-mediated neurological functions. As stated before, GPER is expressed throughout the central and peripheral nervous system of male and female rodents and humans (49). Acute administration of E2 or GPER selective agonists STX or G-1, improved neuron survival rate by 40–45% compared to control in ovariectomized female rats (145). In contrast, G-1 promoted apoptosis of rat embryo cortical astrocytes exposed to oxygen and glucose deprivation, whereas the addition of the GPER antagonist G-15 suppressed this effect, suggesting a direct impact of GPER on the viability of cortical astrocytes (146). Interestingly, administration of G-1 counteracted iron- and ovariectomy-induced memory impairments in female rats (147). Therefore, GPER could be a novel target in treatment of neurodegenerative diseases, such as memory disorders, Alzheimer's disease and ischemic stroke. Although the results from limited studies using selective chemicals of GPER consistently demonstrate a neuronal effect of GPER, it remains to be determined whether GPER truly acts as an E2 receptor. As reported, infusion of G-1 or E2 promoted memory function in ovariectomized female mice, but G-1 activated the c-Jun N-terminal kinase while E2 stimulated ERK1/2. In addition, G15 failed to block the activation of ERK1/2 induced by E2, but infusion of G15 to the dorsal hippocampus impaired memory formation and object recognition (148). These data suggest that the benefits of hippocampal GPER on memory function is not mediated by E2. Thus, the role and precise sites in neurons responsible for GPER action need to be elucidated, which may be achieved by using tissue-specific knockout animal models.

### GPER and Glucose Metabolism

While the classic ERs have been known to play a role in mediating E2 effects on glucose metabolism and metabolic diseases, the metabolic action of GPER remains to be determined. The generation of GPER knockout (GPRKO) mice facilitates our understanding of the physiology role of GPER. Martensson et al. showed for the first time that GPRKO female mice displayed hyperglycemia, impaired glucose tolerance, and reduced body weight and bone growth, whereas GPRKO male mice were metabolically normal (57), thus demonstrating a gender-dependent effects of GPER on glucose homeostasis and animal growth. The potential anti-diabetic effect of GPER was revealed from studying the ERα/β double knockout (DKO) mice treated with streptozotocin (STZ) (149), in which E2/ERα/β signaling was removed, thereby allowing to determine only GPER-mediated action of E2. The results indicated that ovariectomized ERα/β DKO mice were more susceptible to STZ-induced islet apoptosis and diabetes as compared with sham-operated ERα/β DKO mice, but the STZ-induced islet apoptosis and diabetes in ovariectomized ERα/β DKO mice were attenuated by E2 replacement therapy (149), suggesting that E2/GPER signaling is protective against STZ-induced insulin deficient diabetes. Indeed, the authors further demonstrated that female GPRKO mice were predisposed to insulin-deficient diabetes due to increased β-cell apoptosis. In accordance with this *in vivo* result, GPER agonist G-1 directly protected mouse and human islets against oxidative stress-induced apoptosis, and E2 still promoted pancreatic β-cell survival in ERα/β DKO mice exposed to STZ (150). Taken together, these results showed that in the absence of the classical ERα/β, E2 may signal through GPER to protect against STZ-induced islet apoptosis. Consistently, data from several other studies showed that deletion of GPER resulted in a reduced insulin secretion from pancreas, suggesting that GPER indeed plays a role in maintaining metabolic functions via regulating insulin secretion in mice (149, 151, 152). Furthermore, the protective effect of E2 on pancreatic β-cells can be mimicked by GPER agonist, genistein (153). Interestingly, we recently found that deletion of GPER protected female mice from high-fat diet (HFD)-induced obesity and hyperglycemia (154). After 15 weeks of HFD feeding, their blood glucose levels gradually diverged with GPRKO displaying significantly lower fasting and non-fasting blood glucose levels as compared with those in WT while their insulin sensitivity was not different. The reason for these discrepancies are not clear. It should be noted that our study used GPER mice in 129 background in contrast to C57BL/6 GPER mice as used in other studies. Other factors, such as the genetic knockdown or knockout strategy, the breeding strategy, and the environment can have unexpected influence on the phenotypes as well. Of the note, certain maternal and/or experimental diets contain significant amount of phytoestrogens (i.e., soy protein or alfalfa meal) (155), which could modulate the estrogenic activity and therefore could profoundly alter the related outcome of a study given the well-documented various effects of dietary phytoestrogens in rodent models (156, 157).

### GPER and Obesity

While the classical ERs have been well-investigated regarding their roles in mediating E2 effects on fat metabolism and metabolic diseases, little is known about metabolic action of GPER as well as the possible complex interactions among the three ERs in different cell types. E2 and STX, a synthesized non-steroidal compound acting as a GPER selective agonist (158), rapidly attenuated the baclofen response in hypothalamic arcuate POMC neurons in WT, ERαKO, ERβKO, and ERα/β DKO mice, and prevented excessive body weight gain in ovariectomized guinea pigs, suggesting a potential role of GPER in energy metabolism in females (159). Multiple studies have investigated the role of GPER in regulating body weight and fat deposits. The first such study reported an increase in body weight and visceral adiposity in both male and female GPRKO mice as compared with those in WT mice (151). In addition, Davis et al. reported similar observations that KO mice were heavier than the WT littermates fed a standard chow diet (STD), although this difference between female mice occurred 5 weeks later as compared to male mice (51). However, others found no significant effect of GPER on body weight of both female and male mice (52). Data from a recent study showed an increased body weights in both male and female GPRKO mice caused by increased fat mass with enlarged adipocytes when fed a phytoestrogen free low fat diet (51). However, Martensson et al. reported contrary results that female GPRKO mice exhibited slightly lower body weights as compared with WT, whereas no such differences were observed in male GPRKO mice (57). The reasons for these disparate results are not clear. However, these studies were not designed for investigating the roles of GPER in obesity development. As female mice in these studies were used at their young ages and fed a STD, they remain lean without apparent metabolic abnormalities, which therefore are not sufficient to reveal the role of GPER in obesity development that is typically caused by high calorie intake.

We recently performed relatively long-term study with detailed analyses of body weight and body composition of female GPRKO mice either maintained on a STD or exposed to a phytoestrogen-free HFD (154). There were no differences in their body weight, fat mass, and all other measured metabolic phenotypes between WT and GPRKO either male or female mice on a STD. However, after 23 weeks of HFD feeding, female GPRKO mice gained 61% of their starting body weight while WT female mice increased by 85% with no difference in energy intake between two groups. At 20 weeks, the fat mass of WT was 1.8-fold of that in GPRKO mice with only slightly higher lean body mass in GPRKO animals, suggesting that the difference in body weight between GPRKO and WT female mice was primarily due to their fat mass difference. Interestingly, no such differences in metabolic phenotypes were observed between WT and GPRKO male mice fed a HFD. In addition, the inguinal, gonadal, and perirenal fat pads from GPRKO mice weighted less than those in WT female mice, while the pancreas from GPRKO female mice was slightly heavier than WT mice. All the other measured organs weights are similar, suggesting the reduced fat mass in GPRKO female mice was not due to decreased body growth. Our H&E staining of fat sections revealed that GPRKO female mice had smaller adipocytes as compared to WT female mice fed a HFD. While the reasons for these disparities with respect to GPER modulation of body weight gain from past studies are not clear, which could be due to the different methods generating the transgenic animals as reviewed (71), and the variations of the diet compositions, duration, and environment, but overall they indicate that GPER might play a role in regulating lipid metabolism and controlling adiposity.

While how exactly GPER regulates lipid metabolism is still unclear, it was recently shown that that the effect of GPER on fat mass in HFD-fed female mice was not due to a secondary action by which its deletion altered circulating E2 levels or expression of ERα (154), which is believed to play a major role in mediating estrogenic effects on energy homeostasis (160). Both human and rodent white adipose tissue expresses ERα, ERβ, and GPER, suggesting that E2 signaling could occur through both ERs and GPER. Interestingly, it was reported that GPER and ERα inhibit each other's actions in several types of cells (78, 161, 162). In mice, GPER activation inhibits ERα-dependent uterine growth induced by E2 (78). These data suggest that there could also be a “yin-yang” relationship between GPER and ERα in adipose tissue that balances energy metabolism in response to E2. In that regard, activation of ERα by E2 inhibits adiposity, whereas activation of GPER might promote obesity, an intriguing concept that worth investigation.

## Evidence of E2-Independent Effects of GPER

While data from a large body of literature suggest that GPER appears to mediate E2-triggered several intracellular signaling pathways, this evidence was primarily obtained from *in vitro* studies in which cultured cells were often either overexpressed with GPER or absent of endogenous ERα/β and typically exposed to well-above physiological doses of E2. As aforementioned, the estimated binding affinities of E2 to GPER (3–6 nM) (30, 31) are considerably higher as compared with its binding affinities for classical ERs (0.1–1 nM) (87). This raises an interesting question as to whether GPER plays a significant role in mediating various E2 effects *in vivo*, given that circulating E2 levels in young female rodents are only about 7.3–734.2 pM (163–165), depending on the stage of estrous cycle. Indeed, conflicting results regarding the GPER-mediated signaling events in response to E2 continue to emerge, which warrant further investigations as to whether GPER plays a physiological role as an GPER *in vivo*. Knockdown of GPER in MCF-7 cells expressing ERs and GPER had no impacts on E2-induced cAMP production (166). Others demonstrated that transient expression of GPER in MCF-7 cells resulted in a reduction of cell growth in the absence of E2 (167). Based on these results, GPER may not signal in response to the stimulation of E2 at physiologically relevant levels. Intriguingly, the existence of membrane ERs (mERs) (168–171), though with a limited amount at about 3–10% of the classical nuclear ERs (29, 166), further complicated the rapid non-genomic signaling events mediated by E2. Interestingly, G-1 was shown to induce the phosphorylation of ERK1/2 in GPER-negative HEK293 cells stably transfected with a novel membrane associated ERα, ERα-36 (172), a variant of human ERα-66 (168). Moreover, knockdown of ERα-36 in MDA-MB-231 and SKBr3 cells suppressed the phosphorylation of ERK1/2 and intracellular calcium mobilization stimulated by G-1, suggesting that G-1 also recognizes ERα-36, and therefore it may not be specific for GPER. The use of ICI, an ERα antagonist but GPER agonist, and GPER antagonist G-15, in the mouse hippocampal cell lines mHippoE-14 and mHippoE-18 demonstrated that acute E2 treatment protected hippocampal cells from glutamate-induced neurotoxicity and the protective action requires both mERα and GPER (173). In ovariectomized female mice, it was shown that infusion of E2 into the dorsal hippocampus activated ERα and ERβ, leading to ERK1/2 signaling and improved object recognition and spatial memory. However, infusion of G-1 but not E2 activated GPER, which triggered a different cell-signaling mechanism to facilitate hippocampal memory in female mice (148). These results suggest that GPER in the dorsal hippocampus might not act as an ER. In GPER overexpressed COS-7 and CHO cells, E2 only showed specific saturated binding to ERα, but not to GPER (132). Consistently, in primary endothelial cells from ERα/β DKO mice, E2 failed to specifically bind to GPER and activate cAMP, ERK1/2, or PI3K signaling as observed in clonal cancer cells (166).

The reason for these disparate results on the role of GPER in E2 signaling is unclear. Many of these studies were obtained using clonal cell-based experiments, in which cells were manipulated with overexpression of GPER, which may result in ectopic expression of GPER in the cells. Even using the cells that endogenously express GPER, cellular experiment results cannot recapitulate E2 functions in whole body. Taken together, it remains to be determined whether GPER functions as a specific E2 receptor that mediates endogenous E2 effects *in vivo*.

## Concluding Remarks

GPER is an atypical GPCR and has been named as a new ER. While ERα/β have been well-investigated regarding their roles in mediating E2 effects in health and disease, the physiological and/or pathological roles of GPER remain to be determined. The pace of research into the functions of GPER has been accelerating over the past decade with the generation of GPER transgenic mice as well as its selective chemical ligands, which are powerful tools to investigate the physiological and/or pathological role(s) of GPER. Although the results from *in vitro* studies suggest that E2 could activate to trigger various intracellular signaling pathways, and data from animal studies do not exclude GPER as an ER in mediating estrogenic responses, convincing evidence that E2 acts through GPER to elicit significant physiological events *in viv*o is still lacking (58, 132, 174). While the major physiological function of GPER is likely not for promoting reproductive tissue development, increasing evidence suggest that GPER plays a role in body weight regulation and metabolism. However, the clear metabolic effects of GPER and the role of E2 plays in this context need further investigation. In addition, whether GPER counteracts ERα in energy metabolism is an intriguing question that needs to be addressed in future research. Finally, future research should also be aimed at understanding GPER biology in humans, which has been seldom investigated.

## Author Contributions

All authors made substantial contributions to the conception and design of this review paper, drafted the manuscript and revised it critically for important intellectual content, and approval it for publication.

### Conflict of Interest

The authors declare that the research was conducted in the absence of any commercial or financial relationships that could be construed as a potential conflict of interest.
